# Does helminth activation of toll-like receptors modulate immune response in multiple sclerosis patients?

**DOI:** 10.3389/fcimb.2012.00112

**Published:** 2012-08-24

**Authors:** Jorge Correale, Mauricio F. Farez

**Affiliations:** Department of Neurology, Institute for Neurological Research Dr. Raúl Carrea, FLENIBuenos Aires, Argentina

**Keywords:** multiple sclerosis, parasites, helminth, toll-like receptors, soluble egg antigen, MyD88, mitogen activated protein kinase

## Abstract

Multiple sclerosis (MS) is an inflammatory autoimmune demyelinating disease affecting the Central Nervous System (CNS), in which Th1 and Th17 cells appear to recognize and react against certain myelin sheath components. Epidemiological evidence has accumulated indicating steady increase in autoimmune disease incidence in developed countries. Reduced infectious disease prevalence in particular has been proposed as the cause. In agreement with this hypothesis, we recently demonstrated significantly better clinical and radiological outcome in helminth-infected MS patients, compared to uninfected ones. Parasite-driven protection was associated with regulatory T cell induction and anti-inflammatory cytokine secretion, including increased TGF-β and IL-10 levels. Interestingly, surface expression of TLR2, on both B cells and dendritic cells (DC) was significantly higher in infected MS patients. Moreover, stimulation of myelin-specific T cell lines with a TLR2 agonist induced inhibition of T cell proliferation, suppression of IFN-γ, IL-12, and IL-17 secretion, as well as increase in IL-10 production, suggesting the functional responses observed correlate with TLR2 expression patterns. Furthermore, parasite antigens were able to induce TLR2 expression on both B cells and DCs. All functional effects mediated by TLR2 were abrogated when MyD88 gene expression was silenced; indicating helminth-mediated signaling induced changes in cytokine secretion in a MyD88-dependent manner. In addition, helminth antigens significantly enhanced co-stimulatory molecule expression, effects not mediated by MyD88. Parasite antigens acting on MyD88 induced significant ERK kinase phosphorylation in DC. Addition of the ERK inhibitor U0126 was associated with dose-dependent IL-10 inhibition and reciprocal enhancement in IL-12, both correlating with ERK inhibition. Finally, cytokine effects and changes observed in co-stimulatory DC molecules after helminth antigen exposure were lost when TLR2 was silenced. Overall, the data described indicate that helminth molecules exert potent regulatory effects on both DCs and B cells from MS patients through TLR2 regulation.

## Introduction

Multiple Sclerosis (MS) is a chronic autoimmune mediated disease of the Central Nervous System (CNS) characterized by inflammation, demyelination, and axonal loss (McFarland and Martin, 2007). Although its etiology remains unknown, several lines of evidence support the notion that autoimmunity plays a major role in disease development and susceptibility. It is generally accepted that both MS and experimental autoimmune encephalomyelitis (EAE), an animal model resembling MS, involve Th1 and Th17 cells recognizing certain myelin sheath components (Goverman, 2009). Autoimmune diseases such as MS are currently thought to be caused by a combination of individual genetic susceptibility and external environmental factors (Marrie, 2004; Oksenberg and Baranzini, 2010). The genetic component of MS is believed to result from the action of common allelic variants in several genes. However, discordance of MS among monozygotic twins suggests that additional factors, such as environmental modulators could be involved (Oksenberg and Baranzini, 2010).

Several studies implicate infectious and non-infectious environmental factors present during childhood and young adulthood as strong determinants of MS risk, although their exact nature remains unidentified. Microbial infections can act as triggers inducing or promoting autoimmunity, resulting in clinical disease manifestations in genetically predisposed individuals. Alternatively, infections could accelerate sub-clinical autoimmune processes (Christen and von Herrath, 2005; Correale et al., 2006). In contrast to this view, epidemiological and experimental evidence has accumulated in recent decades indicating a steady increase in autoimmune disease incidence in developed countries, which have been associated to a decline in infectious disease prevalence (Bach, 2002). These observations suggest infections may protect rather than induce or accelerate autoimmune diseases like MS.

Several studies both in humans and in animal models have shown helminths to be powerful modulators of host immune responses (La Flamme et al., 2003; Sewell et al., 2003; Jankovic et al., 2006). Following this premise, we recently demonstrated that helminth-infected MS patients showed significantly lower disease activity, compared to uninfected MS subjects (Correale and Farez, 2007). In addition, we provided evidence that autoimmune response down-regulation, secondary to parasite infection in MS patients was mediated through regulatory cell activity, the effect of which extended well beyond classical response to pathogens (Correale and Farez, 2007).

Dendritic cells (DCs) in parasite infected hosts express a range of pattern recognition receptors (PPRs), including Toll-like receptors (TLRs), nucleotide-binding oligomerization domain-like receptors (NLRs), retinoic acid-inducible gene-like receptors (RIG-like receptors), and C-type lectin receptors (CLRs). Upon engagement, signaling cascades are triggered inducing gene expression involved in DC maturation, and in priming and skewing regulatory T cell immune responses (Kane et al., 2004). The fact that helminths can both activate and negatively regulate TLR expression indicates just how a tight control over the immune response these infections exert.

## Basic immunology of MS

Plaques of inflammatory demyelinitation within the CNS are the pathologic hallmarks of MS. Typical features of the acute plaque include ill-defined margins of myelin loss, infiltration by immune cells, and parenchymal edema (Frohman et al., 2006). The constituents of immune cell influx around vessels include T cells, B cells, monocytes, and macrophages. The presence of lymphocytes within plaques and bordering areas suggests inflammatory destruction observed in MS is driven by antigen-specific cells targeting myelin and other CNS components. The determination that EAE can be mediated by CD4+ T cells has promoted intense investigation into potential CD4 T-cell targets in MS. However, how these T cells become abnormally activated against CNS antigens remains unclear. New research has focused on the different roles of CD4+ T cell subsets in MS. Following activation, naïve T cells differentiate into various T cell populations with different effector functions (Boppana et al., 2011). Th1 cells produce pro-inflammatory cytokines such as IFN-γ activating macrophages. Th2 cells secrete anti-inflammatory cytokines such as IL-4. Dysregulation of the balance between Th1 and Th2 cytokines has long been implicated in MS immunopathogenesis. Th17 represents a distinct lineage of effector T cells. IL-23 produced by macrophages and DCs is critical for expansion of Th17 cells synthesizing pro-inflammatory cytokines such as IL-17A and IL-17F (Korn et al., 2009). Interestingly, Th17 to Th1 ratio appears to be a critical determinant of CNS inflammation, with high Th17 to Th1 ratios linked to T cell infiltration and CNS inflammation (Strommes et al., 2008). Cytotoxic CD8+ T cells which are present in MS lesions in significant numbers may also contribute to tissue damage by attacking oligodendrocytes and transecting axons (Babbe et al., 2000; Neumann et al., 2002).

Natural occurring regulatory T cells (CD4+CD25+ FoxP3+; Treg) comprise a small number of CD4+ T cells that have been implicated in MS pathogenesis. Although Treg cell numbers in peripheral blood and CSF appear to be similar in MS patients and in healthy controls, several studies point to defects in the capacity of Tregs from MS patients to suppress myelin-specific T cell activation in the periphery (Viglietta et al., 2004). In addition to CD4+CD25+FoxP3+ Treg, *in vitro* antigen activation leads to Foxp3 expression in CD8+ T cells. This enables CD8+ T cells to also acquire suppressive activity (Correale and Villa, 2010).

Although an autoreactive T cell-mediated immune response has been considered critical for MS pathogenesis, increasing evidence indicates that B cells also play a key role, as indicated by the presence of B cells, plasma cells, immunoglobulins, and complement deposition in autopsy tissues from MS patients, along with immunoglobulin-myelin complex within macrophages (Genain et al., 1999). Intrathecal IgG synthesis, and the recent finding of B-cell lymphoid follicles in the meninges of MS patients with progressive disease, further support this concept (Magliozzi et al., 2007; Franchiotta et al., 2008). In addition, B cells may promote neuroinflammation in MS via secretion of pro-inflammatory cytokines such as TNF-α, and lymphotoxins in the presence of T cell-derived cytokine IFN-γ (Bar-Or et al., 2010). Conversely, B cells are also likely to have immune-suppressive properties. For example, IL-10 secretion by B cells can serve to limit pro-inflammatory autoreactive CD4+ T cell response (Fillatreau et al., 2002).

Immune access to the CNS is generally considered to be restricted. Only by engaging in a critically timed sequence of events can autoreactive lymphocytes enter the CNS compartment. Initially, leukocyte engages in rolling, activation, and arrest along the Blood-Brain Barrier endothelium. This initial step is greatly facilitated by upregulation of endothelial cell adhesion molecules, including ICAM-1 and VCAM-1 (Piccio et al., 2002). Changes in the vascular endothelium could result from pro-inflammatory mediators circulating within the vasculature, including TNF-α, and IFN-γ. The complex sets of molecules that leukocytes depend on for entry into the CNS are integrins, a group of molecules mediating adhesion between cells. Among a panel of leukocyte adhesion receptors, VLA-4 was identified as the crucial factor for encephalitogenic T-cell binding to the endothelium (Yednock et al., 1992). In addition, leukocyte migration to the CNS is increased through the action of chemokines and their receptors, which have been implicated in leukocyte influx into the CNS observed in MS (Holman et al., 2011).

Antigen presenting cells (APCs) also play an important role in the initiation and progression of MS. DCs are a group of APCs which modulate adaptive immune responses, usually present in perivascular spaces, the choroid plexus, and the meninges of healthy brains (Serafini et al., 2006). In MS, DCs among other cell types are recruited to the CNS, representing the major APCs during the secondary phase of cognate interactions with CD4+ T cells within the CNS (Greter et al., 2005). In addition to DCs, microglia, as resident APCs localized in active plaques, play an important role in antigen presentation. Upon activation, microglial cells express greater amounts of Class II MHC and co-stimulatory molecules, promoting pro-inflammatory T cell responses within the CNS (Lassmann et al., 2001).

Although MS was classically described as a disease marked by the loss of myelin, axonal loss has also been observed even in the earliest pathology reports on the disease (Charcot, 1868). Mechanisms for axonal damage are manifold and include: a specific immunological attack on axons (Medana et al., 2001; Neumann et al., 2002); the presence of soluble mediators such as proteases and free radicals released as part of the inflammatory environment present in the CNS of MS patients (Smith and Lassmann, 2002), or lack of neurotrophic factors provided to the axon by oligodendrocytes as a result of demyelination (Frohman et al., 2005). As a consequence of immune-mediated injury to myelin, higher energy demands on demyelinated axons and glutamate-mediated excitotoxicity may impart further unsustainable damage (Werner et al., 2001; Mahad et al., 2009).

**Figure 1:**
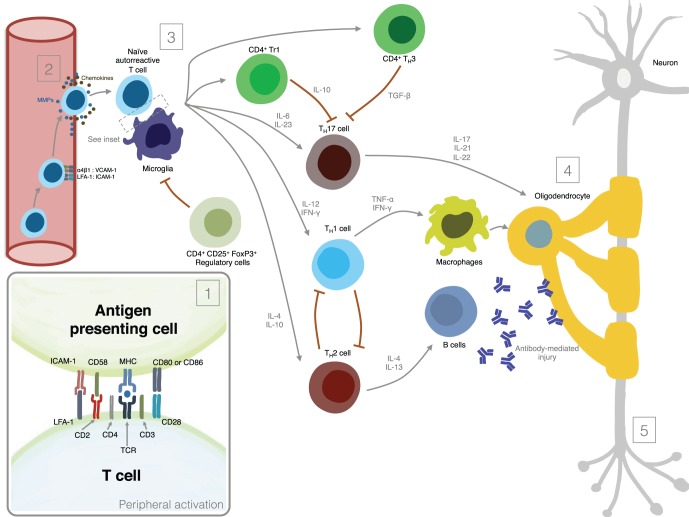
**Mechanisms involved in MS pathogenesis. (1)** Peripheral activation; **(2)** Migration of activated lymphocytes across the Blood-Brain-Barrier; **(3)** Reactivation of lymphocytes in the CNS and secretion of pro-inflammatory mediators; **(4)** Induction of demyelination; **(5)** Induction of axonal damage.

Figure 1 summarizes the most important mechanisms involved in MS pathogenesis.

## The EAE model

Dissecting the pathogenesis of a disease as complex as MS in humans is fraught with many problems, particularly those associated with clinical and genetic heterogeneity. Because brain and spinal cord tissue cannot easily be sampled in MS patients *in vivo*, a number of animal models have been generated to provide insights into the underlying pathology, as well as to identify surrogate biomarkers and therapeutic targets. The basic protocol for inducing EAE involves immunizing animal strains/species using either foreign or self proteins from the white matter of the nervous system, usually myelin basic protein (MBP), proteolipid protein (PLP), or myelin oligodendroglial glycoprotein (MOG; Steinman, 1999). Immunization leads to the development of a disease state in the animals which shows physical and neuropathological similarities to human MS. Clinical course varies from acute monophasic episodes of paralysis in some, to chronic-relapsing neurological episodes and progressive disability in others (Furlan et al., 2009). The episodes correlate with the perivascular mononuclear cell infiltrates in the CNS and in some species and strains extensive myelin destruction and axonal loss (Mix et al., 2010). After an initial period of experimentation with active sensitization, murine models of chronic demyelination progressed to adoptive-transfer technologies, whereby CNS antigen-specific bulk-isolated lymphocytes, T cell lines or T cell clones were administered intravenously into naïve recipients (Brown and McFarlin, 1981). Recently, transgenic murine models have been developed in which disease arises spontaneously, mostly through insertion of myelin-specific human T cell receptors and human histocompatibility complex antigens (Ellmerich et al., 2005; Pöllinger et al., 2009).

Although many features of EAE reflect what is known about the pathophysiology of MS, discrepancies between EAE and MS remain. Extrapolations must therefore be made with caution when predicting what might happen in MS, based on results obtained in EAE (Baker et al., 2011). One critical limitation is that most EAE studies use injection with antigenic material to induce disease onset. This clearly contrasts with the spontaneously arising nature of MS. Transgenic murine models offer a possibility to overcome this limitation. However, these are highly manipulated mice and the exact antigen(s) involved in MS is not yet known, generating limitations in the use of models. Another potential limitation is that EAE research is mostly conducted on highly inbred animals (Handel et al., 2011). Finally, the degree of clinical disease in the EAE model is typically reflected by the lesion load within the spinal cord (Allen et al., 1993). Although some animal strain/species may particularly develop cerebellar lesions, the brain in many EAE models is relatively unaffected (Betelli, 2007). Problematic aspects of animal models are in no way confined to MS alone, and limitations in models should be acknowledged when placing research findings into perspective. Clearly, studies on EAE, despite relative ease, are no substitute for research on bona fide specimens from MS patients.

## Helminth infections modulate immune response and alter clinical course of MS

The best evidence on parasite infections causing immune response modulation in autoimmune diseases derives from animal models. Pre-established infection with the helminth *Schistosoma mansoni*, or pre-treatment of mice with *S. mansoni* ova significantly attenuates clinical course, reduces incidence and delays onset of EAE in mice, and is associated with decreased IFN-γ, TNF-α, IL-12 and nitric oxide production by splenocytes, as well as increased production of IL-10 and TGF-β (La Flamme et al., 2003; Sewell et al., 2003). Moreover, IL-12p40 transcript levels are dramatically reduced in the spinal cord of *S. mansoni* infected animals, correlating with decreased macrophage-infiltrates in brain and spinal cord. Although CD4+ T cell levels are similar during the disease peak, they fall significantly 6 week post-stimulation, suggesting schistosomiasis may affect EAE course by targeting the macrophage compartment, and down-regulating effector functions mediated by T cells. Likewise, infection with *Fasciola hepatica* also exerts bystander suppression of immune responses to autoantigens and attenuates clinical signs of EAE (Walsh et al., 2009). Protection is associated with autoantigen IFN-γ and IL-17 production suppression. Th1 and Th17 response is maintained in IL10^−/−^ mice, but abrogated by *in vivo* TGF-β neutralization, indicating that *F. hepatica* induced TGF-β plays a critical role in the suppression of auto-antigen specific Th1 and Th17 responses. Similarly, in mice infected with *Taenia crassiceps* symptom severity was significantly reduced in 50% of the animals, an effect associated with decreased MOG-specific splenocyte proliferation and IL-17 as well as TNF-α production. Moreover, parasite infection induces an increased production of IL-4 and IL-10, as well as limited leukocyte infiltration in the spinal cord and brain (Reyes et al., 2011). Therefore, the molecular mechanisms responsible for these beneficial effects on disease course seem to differ depending on the helminth species, or helminth-derived products used, and in addition may be host and disease dependent as well.

Recent epidemiological studies demonstrated a dichotomous relationship between worldwide distribution of MS and the parasite *Trichuris trichiura*, a common human pathogen. MS prevalence appears to fall steeply once a critical threshold of *T. Trichiura* (about 10%) is exceeded (Fleming and Cook, 2006). Longitudinal studies including those tracking the prevalence of MS in response to changing sanitation and levels of infection would be very informative to understand the role of helminths in the modulation of MS. In this regard, the increase in MS prevalence in the French West Indies between 1978 and 1994 was correlated with a significant reduction in intestinal parasitism during the same period (Cabre et al., 2005). Although intriguing and provocative the data only demonstrate association, but in no way prove causality. Sanitation may simply be a marker for more fundamental social or environmental factors in developed countries that are directly linked to MS etiology (Fleming and Cook, 2006).

Recently, we identified a group of 12 MS patients (8 women, 4 men; mean age 34 ± 6.8 years) presenting eosinophilia subsequently determined to be caused by mild, asymptomatic intestinal parasitosis, a diagnosis confirmed in all patients by positive stool examination (Correale and Farez, 2007). Three patients were infected with *Hymenolepis nana*, three with *Trichuris trichiura*, three with *Ascacris lumbricoides*, two with *Strongyloides stercolaris*, and one with *Enterobius vermicularis*. At time of eosinophil count, helminth egg load values ranged between 1180 and 9340 eggs/gr of stool. Presence of other endemic parasitoses, including trypanosomiasis, leishmaniasis, amebiasis, giardasis, and toxoplasmosis, were ruled out in this population both by microscopic stool examination and serological testing. Investigations were always negative, excluding simultaneous presence of these infections. Patients were matched and compared to 12 uninfected MS subjects. Following standard medical practice as well as tropical medicine expert recommendations indicating asymptomatic adult patients usually do not require treatment, parasite infected MS patients were not given anti-helminth medication. Both groups were followed for approximately 4.5 years during which time clinical, radiological, and immunological parameters were compared. Parasite-infected MS patients showed significantly lower exacerbation rate, minimal changes in disability, and significantly lower radiological activity compared to uninfected MS individuals.

Parasite-driven protection was associated with induction of regulatory T cells, secreting suppressive cytokines such as IL-10 and TGF-β, as well as CD4+CD25+FoxP3+ T cells displaying significant suppressive function.

These findings provide evidence to support autoimmune down-regulation secondary to parasite infections in MS patients through regulatory T cell action, with effects extending beyond response to the infectious agent. Evidence of regulatory T cell presence during parasite infections is now emerging, offering a potential explanation for mechanisms through which infected hosts exhibit altered immune responses to bystander antigens.

In addition to the development of regulatory T cells, helminths also induce regulatory B cells capable of dampening the immune response through production of IL-10 in MS patients (Correale et al., 2008). The cytokine is essential for regulatory function development in this subset of B cells, and B cells isolated from IL-10 knock-out mice fail to show the protective function (Fillatreau et al., 2002). In agreement with our findings, recent observations indicate that B cells from MS patients exhibit relative deficiency in their IL-10 producing capacity (Duddy et al., 2007). Interestingly, production of IL-10 by B cells is restricted to helminth-infected individuals exclusively. B cells from patients with infections from other parasites such as *Trypanosoma cruzi* exhibit IL-10 levels similar to those in uninfected MS patients, indicating that intracellular parasites are unable to down-modulate harmful autoimmune responses in the way helminths do. Likewise, B cells from *paracoccidioides brasiliensis*-infected patients, although expressing Th2 immune responses after antigen-stimulation of peripheral blood mononuclear cells (PBMC), exhibited B-cell IL-10 production levels similar to those observed in uninfected MS patients, suggesting that increased production of IL-10 by B cells found in helminth-infected MS patients is not determined by the Th2 profile present in these individuals. IL-10 producing B cells isolated from helminth-infected MS patients express MHC Class Ib molecule CD1d, which is involved in antigen presentation and in immunoregulation. Glycolipids presented by CD1d expressing the invariant Vα14 (in mice), or Vα24 (in humans) T cell receptor, activate natural killer T cells, a mechanism found to prevent autoimmune responses in different animal models (Godfrey and Kroneneberg, 2004). Moreover, the cytoplasmic tail of CD1d is linked to signaling cascades associated to IL-10 transcription, suggesting yet another immune regulatory mechanism (Colgan et al., 1999).

Although these results provide clear evidence that persistent helminth infections can induce an anti-inflammatory regulatory network, consequently altering the course of MS, intervention studies are necessary to establish whether a direct link between parasites and the autoimmune response observed in MS exists. Our initial observation on helminth-infected MS patients was extended, and subjects prospectively studied for approximately 7.5 years. After 5.25 years, four patients required anti-helminth treatment. Following drug administration, clinical and radiological activity increased to levels observed in uninfected MS patients (Correale and Farez, 2011). Mechanistic studies revealed that cytokine secretion pattern reverted, with increase in IFN-γ and IL-12 secreting cell numbers, and a robust decline in IL-10 and TGF-β secreting cells as well as in CD4+CD25+Foxp3+ T regulatory cells. These observations provide clear evidence that chronic helminth infections induce anti-inflammatory networks, dampening inflammatory reactions occurring in autoimmune diseases. Based on the observation that increased production of IL-10 by CD4+ T cells and B cells after helminth infection induced modulation of EAE and MS, it seems reasonable to postulate recombinant IL-10 use (rIL-10) for MS patient treatment. Increased IL-10 expression in CNS during recovery from EAE-induced brain inflammation, suggests IL-10 presence is, however, required within the target organ for disease remission to occur (Kennedy et al., 1992). Because of their short half-life, cytokines need to be administered frequently and in large doses to provide therapeutic concentrations at the target organ level, and systemic administration of rIL-10 for treatment of EAE has yielded contradictory results. Intravenous injection of rIL-10 has exacerbated, rather than suppressed EAE (Cannella et al., 1996). In contrast, other studies have shown that peripheral IL-10 treatment can prevent EAE, when administered systemically during the priming phase of the disease (Rott et al., 1994). Should rIL-10 be of therapeutic benefit, then it would need to be administered once the autoagressive response has been generated. IL-10 also, significantly inhibited the effector phase of EAE when administered locally in the CNS using a retrovirus vector (Croxford et al., 2001), providing an optional manner to maintain effective levels of IL-10 when frequent injection is not feasible. These results suggest that although rIL-10 has the potential to prevent CNS inflammation, distribution, and timing of its production may explain its variable effectiveness (O'Garra et al., 2008). Overall it is possible to conclude that systemic administration of rIL-10 may yield suboptimal efficacy, while targeted and persistent delivery of this cytokine in the CNS may be highly effective.

## Expression of TLR in MS patients

TLRs are type I transmembrane glycoproteins composed of extracellular and intracellular signaling domains (Gay and Gangloff, 2007). They are expressed in different combinations on many cells of the immune system, at the cell surface and along endosomal membranes. Extracellular TLR domains have reiterated leucine-rich modules bearing pathogen-associated molecular patterns (PAMPs) able to recognize a wide range of microbial products, thus providing a link between innate and adaptive immunity (Akira, 2003; Kawai and Akira, 2005). The microbial products constituting PAMPs that bind to TLR can be lipids, lipopeptides, proteins, or nucleic acids. Initially, these exogenous PAMPs were described in the context of infection, but endogenous ligands such as self-molecules from apoptotic cells can also trigger inflammation. In particular endogenous heat-shock proteins, extra-cellular matrix fragments, fibrinogen, and self RNA and DNA can bind to TLRs (Yu et al., 2010). Endogenous RNA and DNA are able to activate TLR7 and TLR9 if they enter endosomal compartment, inducing production of pro-inflammatory cytokines by plasmocytoid DCs (Chi and Flavell, 2008). In mammals, specific combinations of TLRs are expressed on immune cells such as DCs, macrophages, mast cells, and to a lesser degree in fibroblasts, epithelial cells, endothelial cells, and also in T and B cells.

The innate immune system plays an important role both in the initiation and progression of MS influencing the effector function of T and B cells (McFarland and Martin, 2007; Boppana et al., 2011). In particular, recent studies have shown that TLRs modulate MS as well as EAE (Monney et al., 2002; Weiner, 2004; Racke and Drew, 2009), and several endogenous TLR ligands have been recently identified in the MS lesions (Lassmann, 2008). The need for the use of Complete Freund's adjuvant (CFA) containing mycobacteria to induce EAE suggests TLR signaling may be important for the induction of encephalitogenic Th17 cell responses and EAE. TLR4 recognizes LPS subsequently inducing a pro-inflammatory Th1 response (Marta et al., 2009). Unexpectedly, lack of TLR4 exacerbates Myelin Oligodendrocyte Glycoprotein (MOG)-induced EAE (Marta et al., 2008). TLR4^−/−^ mice exhibited marked increase in IL-6 and IL-23 expression by splenic myeloid DCs, increased frequency of splenic Th17 cells, and increased levels of serum IL-17. In contrast, MOG-specific Th1 responses are impaired in TLR4^−/−^ mice. This could explain the aggravated Th17 cell response in TLR4^−/−^, because Th1 cells and IFN-γ inhibit Th17 cell differentiation *in vitro* (Harrington et al., 2005).

Mycobacteria are rich in unmethylated CpG DNA that binds to TLR9. The role of TLR9 in EAE has therefore been investigated. Initial experiments demonstrated that TLR9^−/−^ mice exhibited ameliorated symptoms of MOG_35–55_ induced EAE (Prinz et al., 2006). In contrast, other authors demonstrated that TLR9^−/−^ mice exhibited exacerbated symptoms of MOG-induced EAE (Marta et al., 2009). Pro-inflammatory cytokine expression by splenic myeloid DC and T cells is similar in TLR9^−/−^ and wild-type mice, suggesting cells other than myeloid DCs or T cells are responsible for these findings (Marta et al., 2009). Several putative explanations for exist for these conflicting results: first, a net anti-inflammatory effect of TLR9 signaling, despite its role in pathogenic antibody production. Second, induction of cross-tolerance upon repeated stimulation of one TLR family. Finally, induction of tolerance mediated by TLR9 (Marta et al., 2009).

**Figure 2:**
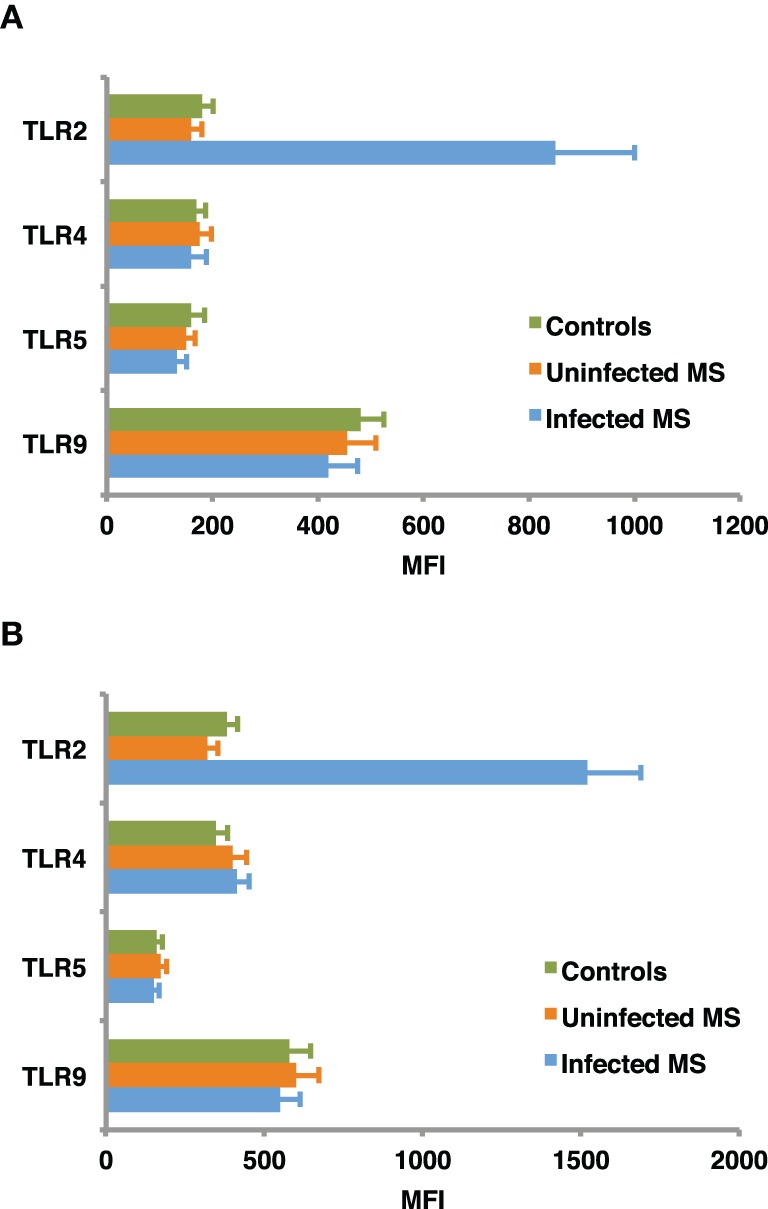
**Baseline expression of TLRs on B cells (A) and dendritic cells (B).** CD19+ B cells and dendritic cells, were isolated from peripheral blood mononuclear cells using specific isolation kits according manufacturer's instructions to a purity of 95–98%. TLR2, TLR4, TLR5, and TLR9 expression was assessed in B cells and monocyte derived dendritic cells from helminth-infected MS patients, uninfected MS patients, and healthy controls, using flow cytometry. Mean fluorescence intensity (MFI) of TLR expression was corrected after subtracting MFI of the control antibody isotype. TLR2 expression in infected MS patients was found to be significantly higher (*p* < 0.001) than in uninfected MS patients or healthy controls. In contrast, MFI of TLR4, TLR5, and TLR9 did not differ between groups. Data represent mean values ± SEM from 12 subjects for each group.

**Figure 3:**
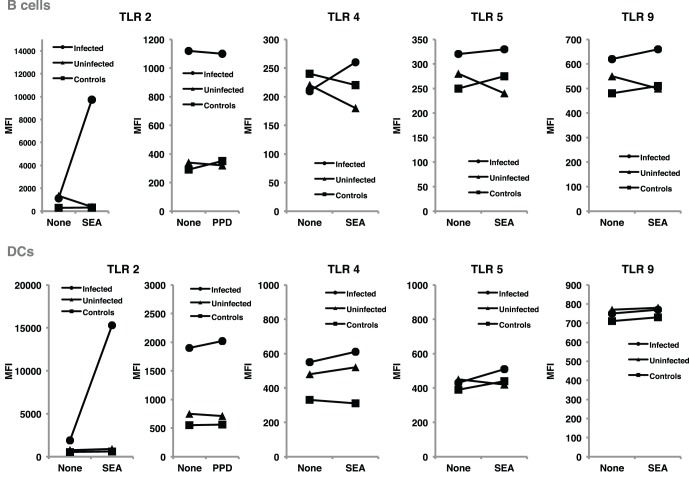
**SEA upregulated TLR2 expression on B cells and dendritic cells, from helminth-infected MS patients.** B cells and dendritic cells were cultured for 48 h in the presence and in the absence of SEA, or PPD used as control antigen. TLR expression was evaluated using flow cytometry, and results expressed as mean fluorescence intensity. Following exposure to SEA, TLR2 was markedly up-regulated in B cells and dendritic cells from helminth-infected MS patients. In contrast, no significant changes were observed in TLR2 expression after PPD exposure. Likewise, exposure to SEA did not affect TLR4, TLR5, or TLR9 expression on B cells or dendritic cells. Data illustrate MFI from a representative patient. Similar results were observed in 11 additional patients.

TLR regulation may be altered by helminth parasites, affecting its function and levels of expression. Our group has provided evidence suggesting that surface expression of TLR2 on both B cells and DCs is significantly higher in helminth-infected MS patients (Figure 2) and that exposure of either cell population to soluble egg antigen (SEA) obtained from *S. mansoni* resulted in significant upregulation of TLR2 in helminth-infected MS patients, but not in uninfected individuals (Figure 3; Correale and Farez, 2009). It is well known that a variety of pathogens can cause increased TLR expression. Some authors have found results similar to our observations, indicating higher expression of TLR2 on PBMCs, and enhanced response to the Pam3Cys ligand in *Plasmodium falciparum*-infected children, compared to uninfected counterparts (Hartgers et al., 2008). Other reports, however, show down-regulation of TLR expression to be an important immune evasion strategy induced by some protozoan parasites, and also by filarial parasites (Babu et al., 2005). Interestingly, cells from uninfected subjects did not exhibit alterations in TLR expression. It is feasible that TLRs become desensitized in uninfected individuals and, consequently, SEA is not effective in inducing changes in expression levels. Supporting this notion, one study has shown that gut TLRs are constitutively non-responsive to the TLR4 ligand LPS (Lotz et al., 2006). Factors such as total body parasite burden and infection duration may also play an important role in determining TLR expression levels. Alternatively, differences in polymorphisms within TLR genes or in signaling protein pathways may alter responses to different infectious agent stimuli (Lorenz et al., 2000). Finally, lack of SEA effect on uninfected cells may be related to engagement of a second receptor, absent in uninfected cells.

TLR are expressed on CNS cells and can also influence local immune responses. There is marked increase in TLR expression both in MS and EAE brain lesions, and cerebro-spinal fluid mononuclear cells (Bsibsi et al., 2002). Both murine and human microglia mount specific activation programs suited to different TLR signals, particularly TLR9, linking innate and adaptive immune responses at sites where CD4+ T cells undergo activation or proliferate (Chearwae and Bright, 2008; Marta et al., 2009). Conversely, it was recently shown that TLR3 triggers neuroprotective responses in astrocytes, controlling the growth of axons and neuronal progenitor cells (Bsibsi et al., 2010). Overall, it is clear that TLR are potent modulators of microglia cells and macrophage activation programs in the CNS, as well as of neuroprotective responses. Table 1 summarizes the main biological effects of TLRs on the course of EAE and MS.

**Table 1:** **Biological effects of TLR on EAE and MS**.

**TLR/ligand**	**Target cell**	**Biological effects**	**EAE**	**MS**
TLR2/SEA	B cells and DCs	Upregulation of TLR2; induction of tolerogenic DCs Increased production of eotaxin, IL-5, and IL-13, TGF-β, and IL-10		
		Suppression of IL-12, IL-6, IL1-β, and TNF-α Down regulation of LPS-induced co-stimulatory molecules on DCs	+	+
		Induction of IL-10 producing Treg cells		
TLR2 and TLR4/HMGB1	Macrophages and microglia	Increased expression of IL-23		+
TLR2/zymosan	DCs	Induction of tolerogenic DCs, increased production of IL-10 and TGF-β		
		Decreased production of IL-6 and IL-12	+	
TLR3	Astrocytes	Triggers neuroprotective responses, controlling the growth of axons, and neuronal progenitor cells	+	+
TLR3	Microglia	Th1 cell polarization, increased production of IFN-γ concomitant with increased CD4+ T cell death	+	
TLR4/LPS	T cells	Induction of pro-inflammatory Th1 responses	+	
	Myeloid DCs	TLR4–/– mice shown exacerbation of MOG-induced EAE		
		Increased production of IL-6 and IL-23 by myeloid DCs	+	
		Increased number of Th17 cells and serum levels of IL-17		
		Impaired Th1 responses		
TLR9/CpG DNA	Myeloid DCs	TLR9–/– mice exhibited ameliorated symptoms of EAE	+	
	Other cells different from DCs and T cells	TLR9–/– mice exhibited exacerbated symptoms of EAE	+	
TLR9	Microglia	CD4+ T cells undergo activation and proliferation	+	+

DCs, Dendritic cells; HMGB1, High mobility group box chromosomal protein; SEA, Soluble egg antigen.

In addition to TLRs, DCs express a variety of receptors specialized in the recognition of glycans, of which CLRs are the most important (Weis et al., 1998). Recent data suggests glycan-induced CLR signaling modulates inflammatory responses by enhancing or suppressing TLR-mediated responses. DC-SIGN especially shows interesting properties associated with Th2 polarization, suppression of inflammation, and induction of regulatory T cell populations (Bergman et al., 2004). Mannosylated Mycobacterial cell wall components suppressed LPS-induced TLR4 signaling, indicating crosstalk between DC-SIGN and TLR4 (Geijtenbeek et al., 2003). In addition, one particular DC-SIGN has been shown to be specific for the Lewis^x^ determinant present in *S. mansoni*, and may participate in the effects it has shown on the immune response (Kuijk and van Die, 2010). Recently, another C-type lectin, DCIR was described as suppressing TLR activation, by inhibiting TLR-8 mediated IL-12 and TNF-α production by DCs (Meyer-Wentrup et al., 2009). Physical interaction between CLRs and other receptors at the cell surface may lead to receptor cross-linking and further modification of intracellular signaling (Kuijk and van Die, 2010). These observations suggest helminth glycans may target C-type lectins on DCs, further modulating their immunological functions.

## Molecular mechanisms involved in TLR binding and signaling pathways

TLR signaling has been extensively investigated in the last several years. The cytoplasmic domain of TLR, termed Toll/interleukin-1 receptor (TIR), is highly conserved and functions as binding site for downstream adaptor molecules. Signaling TLR involves a variety of adaptor proteins (Watters et al., 2007), the most common being the myeloid differentiation marker 88 (MyD88) signaling through all TLRs, except TLR3 (O'Neill and Bowie, 2007). Activated TLR recruit MyD88, leading to subsequent activation of downstream targets, including nuclear factor kappa B (NF-κB), mitogen activated protein kinases (MAPKs) (p38, JNK, and ERK1/2), KB kinase inhibitor (IKK), and interferon regulatory factors (IRF; West et al., 2006). After endocytosis into endosomes, both TLR3 and TLR4 signal through the TIR-domain-containing adapter, inducing IFN-β (TRIF) to activate downstream signaling. In addition, MyD88 adaptor-like (Mal, also called TIRAP) is recruited by TLR2 and TLR4, and so far, its function appears to be to subsequently stabilize MyD88 in the complex acting as a bridge (O'Neill, 2008; Beutler, 2009).

TLR2 has been implicated as a potential receptor for SEA immunomodulatory activities (Table 1), after observing that schistosome-derived lipids elicited tolerogenic DCs, triggered eotaxin, IL-5, and IL-13 production, and induced IL-10 producing regulatory T cells via TLR2 recognition (van der Kleij et al., 2002; Magalhaes et al., 2010). Moreover, during experimentally-induced infections, TLR2 absence was associated with enhanced Th1 and reduced Th2 responses, and increasing disease severity (Layland et al., 2007). It is generally accepted that schistosoma-derived lipids signal via TLR2, whereas dsRNA from schistosomal eggs activates TLR3 (Aksoy et al., 2005).

**Figure 4:**
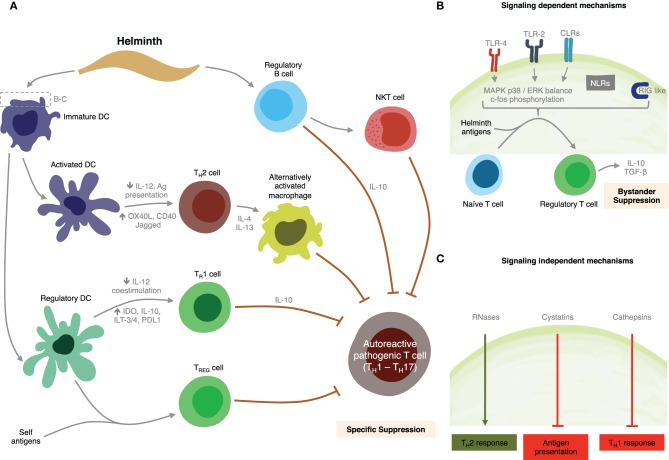
**Summary of putative mechanisms through which helminths could modulate MS. (A)** Helminth-derived products influence the development of specific immunosuppressive mechanisms through interactions with dendritic cells, and B cells. **(B)** Signaling-dependent mechanisms generated by helminth products. Binding of carbohydrates or glycolipid helminth-derived products trigger MAPK cascades, resulting in c-Fos phosphorylation. **(C)** Helminth-derived products favor Th2 responses in dendritic cells by signaling-independent pathways mediated by RNAses, cystatins, and cathepsins.

We have shown that SEA and TLR2 agonists modulate intracellular pathways resulting in suppression of IL-12, IL-6, IL-1β, and TNF-α DC production, and activation of TGF-β, as well as IL-10 production by both DCs and B cells through a MyD88-dependent pathway (Correale and Farez, 2009). We also found SEA down-regulated expression of LPS-induced co-stimulatory molecules on DCs in a MyD88-independent manner (Correale and Farez, 2009). These patterns of DC response are similar to those observed after stimulation using *Giardia* extracts (Kamda and Singer, 2009). Collectively, these observations suggest SEA is able to regulate multiple pathways downstream of TLRs, and that schistosoma contribute to maintain an anti-inflammatory milieu through multiple mechanisms. Supporting these findings, previous studies have demonstrated that mice with B-cell specific deletions of TLR2 and TLR4 or of the TLR adaptor MyD88, do not recover from EAE, indicating TLR participate directly in regulatory functions exerted by B cells (Lampropoulou et al., 2008). Likewise, ES-62 a glycoprotein from the rodent nematode *Acanthonema vitae* inhibited both B and T cell activation through the MyD88-dependent TLR4 pathway (Goodridge et al., 2005). However, and in contrast to these observations, studies using DCs derived from TLR2^−/−^ and TLR4^−/−^ mice have demonstrated that TLR2 and TLR4 are not required for SEA-pulsed DCs to induce anti-inflammatory responses in naïve mice, suggesting other receptors, such as C-type lectins, may be implicated in DCs response capacity to SEA. Discrepancies between investigations may be due to the presence of different moieties in SEA preparations or, alternatively, differences in parasite Ag recognition between human and mouse TLR2s (Kane et al., 2008). Figure 4 summarizes potential mechanisms for immune regulation mediated by helminths as discussed above.

One of the major signaling cascades triggered as a result of TLRs engagement is the MAPK pathway. Differential activation of MAPK p38 and extracellular-signal-regulated kinase (ERK) in DCs has been associated with varying levels of cell maturation and cytokine production. p38 is thought to be important in DC-maturation, and pro-inflammatory T cell responses, whereas ERK activation has more often been associated with anti-inflammatory and Th2 responses (Agrawal et al., 2003; Correale and Farez, 2009). Recent analysis of signaling pathways induced in DCs by carbohydrates from *Schistosoma* eggs has indicated that TLR2 agonists induce increased phosphorylation of the MAPK ERK1/2. Neither stimulus showed effect on p38 or JNK1/2 phosphorylation, however. Interestingly, ERK 1/2 activity blockade using the specific inhibitor U0126, significantly enhanced IL-12 and inhibited IL-10 production induced by TLR2 agonists (Agrawal et al., 2003; Correale and Farez, 2009). In contrast, TLR4 and TLR5 agonists have been shown to preferentially induce IFN-γ and IL-12 via a mechanism involving p38 and JNK1/2 (Rincon et al., 1998; Agrawal et al., 2003; Marthur et al., 2004). ERK 1/2 signaling induced by TLR2 agonists and SEA results in c-Fos phosphorylation (Agrawal et al., 2003). Silencing of c-Fos causes marked IL-12 production, behaving like a Th1 stimulus, indicating that the bias toward IL-10 production observed after TLR2 or SEA stimulation is mediated by c-Fos phosphorylation (Agrawal et al., 2003). It is unclear at present whether SEA stimulation affects other signaling cascades, such as PI3K/Akt pathway. Identification and characterization of signaling molecules involved in MyD88-indpendent pathways remain a challenge for the future.

Overall, these findings provide evidence that helminth parasites and their antigens modulate TLR expression and consequently the innate immune response, preventing host tissue damage. TLR expression and function can, however, have bystander effects such that responses to non-helminth pathogens are also dampened inducing a protective effect against autoimmune diseases such as MS. These protective effects are mediated mainly by suppression of pro-inflammatory cytokines and activation of IL-10, as well as by expansion of CD4+CD25+ FoxP3+ Treg cells. Furthermore, these mechanisms are dependent on ERK 1/2 signaling resulting in the phosphorylation of the early transcription factor c-Fos.

## Parasite antigens may modulate DCs through PPRs-signaling independent mechanisms

Evidence is emerging that helminth manipulation of DCs may also be mediated by mechanisms other than receptor-mediated signaling events, for example, by the enzymatic activities of helminth-derived products (Figure 4). Helminth parasites are known to release a wide variety of enzymatically active products that are thought to play an important role in establishing and maintaining infection, by contributing to the degradation of soluble antiparasitic molecules or through impairment of innate immune cells (Everts et al., 2010). Omega-1 a glycoprotein derived from schistosoma eggs specifically primes DCs to drive polarized Th2 responses (Everts et al., 2009). Although the target receptor is currently unknown it has been suggested that its ribonuclease activity is involved in this effect. Furthermore, a number of studies have documented the potent effects of cystatins, a class of molecules expressed by filiarial nematodes, on host immune responses. Helminth cystatins from *A. vitae, B. Malayi, N. braisliensis*, and *O. volvolus* interfere with DC antigen presentation by blocking host cysteine protease activity (Klotz et al., 2011). Bm-CPI-2, a cystatin from *B. Malay* was described as interfering with antigen processing, which led to a reduced number of epitopes presented to T cells *in vitro* (Manoury et al., 2001). Recombinant AvCystatin, a cystatin derived from *A. vitae* is recognized by macrophages and upon uptake induces phosphorylation of the mitogen-activated protein kinase signaling pathway ERK1/2 and p38 in macrophages, leading to induce IL-10 secretion (Hartmann et al., 1997). Finally, helminth pathogens express cysteine proteases, termed cathepsines, which cause immune deviation by suppressing Th1 immunity. Based on observations of Der p1, one of the major allergens in house dust, it is conceivable for helminth-derived proteases to have the potential to favor Th2 polarization through functional modulation of DC (Hammad et al., 2001). This mechanism is probably mediated by the inhibition of TRIF-dependent signaling via degradation of TLR3 within the endosome (Donelly et al., 2010).

## Helminth-derived immunomodulatory molecules

Whilst the exact signaling pathways by which immune responses are initiated remains obscure, helminth products seem able to interact with host cells and interfere with their function via distinct mechanisms including Treg, B cells and induction of regulatory cytokines such as IL-10 and TGF-β as discussed in the previous sections.

Several studies led to the discovery of specific immunomodulatory helminth-derived molecules and products inducing microenvironments beneficial for parasites while at the same time blocking autoimmune responses (Adisakwattana et al., 2009; Danilowicz-Luebert et al., 2011). Many helminth molecules are homologous to mammalian cytokines. For example two TGF-β homologs found in *Brugia* species, *Bm-tgh-1* and *Bm-tgh-2* have been well characterized (Gomez-Escobar et al., 2000). Similarly, *S. mansoni* male worms express a member of the TGF-β receptor family known as SmRK-1 (Beall and Pearce, 2001), and a product secreted by *H. polygyrus* contains remarkable TGF-β-like activity inducing Foxp3 expression in naïve T cells, modulating immune function (Grainger et al., 2010).

Another well characterized helminth molecule is ES-62, a glycoprotein from the rodent nematode *A. vitae* which exerts several immunomodulatory effects including inhibition of B- and T-lymphocyte proliferation, inhibition of IFN-γ, IL-12, and IL-17 secretion, and inhibition of maturation of naive DCs priming T cells (Harnett and Harnett, 2009). These effects seem to be dependent on phosphorylcholine moieties present on glycoproteins secreted by the parasites. These immunological effects have been observed during therapeutic administration of ES-62 after collagen-induced arthritis induction in mice, resulting in significant reduction of disease severity and progression (Adisakwattana et al., 2009; Mastisz et al., 2011). Likewise, when rDiAg, a product from the filarial parasite *Dirofilaria immitis*, is administered to NOD mice it completely prevents insulinitis and diabetes onset (Imai et al., 2001). Furthermore, *S. mansoni*-specific lysophosphatidylserine activates TLR2 at the cell surface of DCs, generating mature DCs with the ability to promote IL-10 secreting regulatory T cell development. Similarly, other products derived from *S. mansoni* modulate the immune response. Sm16 activates the production of IL-1 receptor antagonists from human keratinocytes, inducing secretion IL-10 and down-regulating ICAM-1 expression. Lacto-N-fucopentose III (LNFP III) contained in Schistosoma eggs is an oligosaccharide that induces IL-10 producing B1 cells in mice (Velupillai et al., 1997). SmCKBP secreted from *S. mansoni*, *S. haematobium*, and *S. japonicum* eggs, suppresses inflammation by binding to different chemokine subfamilies (Adisakwattana et al., 2009). Finally, products from *Ascaris suum* have also been described to contain immunomodulatory properties. The administration of PAS-1 a 200 kDa protein from *A. suum* to mice that have been injected with LPS results in significant decrease in neutrophil migration, increase in IL-10 production, as well as suppression of IL-6, TNF-α, and IL-1β (Oshiro et al., 2005).

## Concluding remarks

Parasites inhabit immune-competent hosts for long periods and can therefore develop modulatory molecules, generating strong anti-inflammatory responses that on one hand may restrict host tissue damage, but on the other can also enhance parasite survival. Several studies have demonstrated that chronic helminth infections in particular, but not acute infections, are associated with the expression of regulatory networks necessary for down modulating autoimmune responses to harmless antigens. In addition, worm glycans may play a role in helminth-induced biasing of the immune response, contributing to the observed protection against autoimmune diseases. Significant interest has arisen in defining and characterizing specific helminth molecules with important immunomodulatory capacities as targets for therapeutic applications in the treatment or prophylaxis of autoimmune diseases. It would be dangerous, however, to ignore the fact that this regulatory environment may hamper essential and necessary responses to other antigens, vaccinations, and life-threatening pathogens. The concern exists because wherever helminth infections are prevalent, so too are dangerous microbial agents such as *Mycobacterium tuberculosis*, human immunodeficiency virus, and malarial parasites. Impaired Th1 responses to tetanus toxoid immunization (van Riet et al., 2008) and against influenza virus in Schistosoma and Onchocerca infected patients (van Riet et al., 2007, 2008) have also been reported, as well as reduced responses to BCG vaccination and to the cholera vaccine during intestinal helminth infection (Elias et al., 2008). Another area of specific safety concern is the possibility of helminths becoming pathogenic in patients with impaired immunity like those treated with immune suppressive medications. For example, S. Stercolaris could multiply and the infection become fulminant when hosts are treated with glucocorticoids (Elliot and Weinstock, 2009). Improving current knowledge of these effects is therefore essential for the development of novel therapeutic approaches for the treatment of autoimmune diseases such as MS. It would be critical to focus on the investigation of immunomodulatory helminth-derived molecules to selectively induce regulatory immune responses, and avoid any possible side effects of natural worm infections. Serious efforts should also be made to generate antigen-specific immunoregulation in order to circumvent possible interference with essential responses to other antigens.

### Conflict of interest statement

Jorge Correale is a board member of Merck-Serono Argentina, Novartis Argentina, Biogen-Idec LATAm, and Merck Serono LATAM. Dr. Correale has received reimbursement for developing educational presentations for Merck-Serono Argentina y LATAM, Biogen – Idec Argentina, and TEVA-Tuteur Argentina as well as professional travel accommodations stipends. Mauricio F. Farez has no conflict of interest to declare.

## References

[B1] Adisakwattana P. Saunders S. P. Nel H. J. Fallon P. G. (2009). Helminth-derived immunomodulatory molecules, in Pathogen-derived Immunomodulatory Molecules, ed Fallon P. G. (New York, NY; Landes Bioscience and Springer Science), 95–107 10.1007/978-1-4419-1601-3_8 20054978

[B2] Agrawal S. Agrawal A. Dougthy B. Gerwitz A. Belnis J. Van Dyke T. Pulendran B. (2003). Different Toll-like receptor agonists instruct dendritic cells to induce distinct Th responses via differential modulation of extracellular signal-regulated kinase-mitogen-activated protein kinase and c-Fos. J. Immunol. 171, 4984–4989 14607893 10.4049/jimmunol.171.10.4984

[B3] Akira S. (2003). Mammalian Toll-like receptors. Curr. Opin. Immunol. 15, 5–11 10.1016/S0952-7915(02)00013-4 12495726

[B4] Aksoy E. Zouain C. S. Vanhoutte F. Fontaine J. Pavelka N. Thiebelemont N. Willems F. Ricciardi-Castiglioni P. Goldman M. Capron M. Ryffel B. Trottein F. (2005). Double-stranded RNAs from the helminth parasite schistosoma activate TLR3 in dendritic cells. J. Biol. Chem. 208, 277–283 10.1074/jbc.M411223200 15519998

[B5] Allen S. J. Baker D. O'Neill J. K. Davison A. N. Turk J. L. (1993). Isolation and characterization of cells infiltrating the spinal cord during the course of chronic relapsing experimental allergic encephalomyelitis in the Biozzi AB/H mouse. Cell. Immunol. 146, 335–350 10.1006/cimm.1993.1031 8174174

[B6] Babbe H. Roers A. Waisman A. Lassmann H. Goebels N. Hohlfeld R. Friese M. Schröder R. Deckert M. Schmidt S. Ravid R. Rajewski K. (2000). Clonal expansion of CD8+ T cells dominate the infiltrate in active multiple sclerosis lesions as shown by micromanipulation and single cell polymerase cell reaction. J. Exp. Med. 192, 393–404 10934227 10.1084/jem.192.3.393 PMC2193223

[B7] Babu S. Blauvelt C. P. Kumaraswami V. Nutman T. B. (2005). Diminished expression and function of TLR in lymphatic filariasis: a novel mechanism of immune dysregulation. J. Immunol. 175, 1170–1176 16002719 10.4049/jimmunol.175.2.1170

[B8] Bach J. F. (2002). The effect of infections on susceptibility to autoimmune and allergic diseases. N. Engl. J. Med. 347, 911–920 10.1056/NEJMra020100 12239261

[B9] Baker D. Gerritsen W. Rundle J. Amor S. (2011). Critical appraisal of animal models of multiple sclerosis. Mult. Scl. J. 17, 647–657 10.1177/1352458511398885 21372117

[B10] Bar-Or A. Fawaz L. Fan B. Darlington P. J. Rieger A. Ghorayeb C. Calabresi P. A. Waubant E. Hauser S. Zhang J. Smith C. H. (2010). Abnormal B-cell cytokine responses trigger of T-cell mediated diseases in MS? Ann. Neurol. 67, 452–461 10.1002/ana.21939 20437580

[B11] Beall M. J. Pearce E. J. (2001). Human transforming growth factor-β activates a receptor serine/threonine kinase from the intravascular parasite *Schistosoma mansoni*. J Biol. Chem. 276, 31613–31619 10.1074/jbc.M104685200 11406634

[B12] Bergman M. P. Engering A. Smits H. H. van Vliet S. J. van Bodegrave A. A. Wirth H. P. Kapsenberg M. L. Vandenbroucke-Grauls C. M. van Kooyk Y. Appelmelk B. J. (2004). *Helicobacter Pylori* modulates the T helper cell 1/T helper cell 2 balance through phase-variable interaction between lipopolysaccharide and DC-SIGN. J. Exp. Med. 200, 979–990 10.1084/jem.20041061 15492123 PMC2211851

[B13] Betelli E. (2007). Building different mouse models for human MS. Ann. N.Y. Acad. Sci. 1103, 11–18 10.1196/annals.1394.021 17376825

[B14] Beutler B. (2009). Microbe sensing, positive feedback loops, and the pathogenesis of inflammatory diseases. Immunol. Rev. 227, 248–263 10.1111/j.1600-065X.2008.00733.x 19120489 PMC2713013

[B15] Bsibsi M. Bajramovic J. J. Vogt M. H. van Duijvenvrooden E. Bagaht A. Persoon-Deen C. Tielen F. Verbeek R. Huitinga I. Ryffel B. Kros A. Gerritsen W. H. Amor S. van Noort J. M. (2010). The microtubule regulator stathmin is an endogenous protein agonist for TLR3. J. Immunol. 184, 6929–6937 10.4049/jimmunol.0902419 20483774

[B16] Bsibsi M. Ravid R. Gveric D. van Noort J. M. (2002). Broad expression of Toll-like receptors in the human central nervous system. J. Neuropathol. Exp. Neurol. 61, 1013–1021 12430718 10.1093/jnen/61.11.1013

[B17] Boppana S. Huang H. Ito K. Dhib-Jalbut S. (2011). Immunologic aspects of Multiple Sclerosis. Mt. Sinai J. Med. 78, 207–220 10.1002/msj.20249 21425265

[B18] Brown A. M. McFarlin D. E. (1981). Relapsing experimental allergic encephalomyelitis in the SJL/J mouse. Lab. Invest. 45, 278–284 6792424

[B19] Cabre P. Signate A. Olindo S. Merle H. Caparros-Lefebvre B. Bera O. Smadja D. (2005). Role of return migration in the emergence of multiple sclerosis in the French West Indies. Brain 128, 2899–2910 10.1093/brain/awh624 16183661

[B20] Cannella B. Gao Y. L. Brosnan C. Raine C. S. (1996). IL-10 fails to abrogate experimental autoimmune encephalomyelitis. J. Neurosci. Res. 45, 735–746 10.1002/(SICI)1097-4547(19960915)45:6<735::AID-JNR10>3.0.CO;2-V 8892085

[B21] Charcot M. (1868). Histologie de la sclerose en palques. Gaz. Hosp. 141, 554–555

[B22] Chearwae W. Bright J. J. (2008). 15-deoxy-Delta (112-14)-prostaglandin J(2) and curcumin modulate the expression of Toll-like receptors 4 and 9 in autoimmune T lymphocyte. J. Clin. Immunol. 28, 558–570 10.1007/s10875-008-9202-7 18463970

[B23] Chi H. Flavell R. A. (2008). Innate recognition of non-self nucleic acid. Genome Biol. 9, 211.1–211.6 10.1186/gb-2008-9-3-211 18341708 PMC2397494

[B24] Christen U. von Herrath M. G. (2005). Infections and autoimmunity – good or bad? J. Immunol. 174, 7481–7486 15944245 10.4049/jimmunol.174.12.7481

[B25] Colgan S. P. Hersberg R. M. Furuta G. T. Blumberg R. S. (1999). Ligation of intestinal epithelial CD1d induces bioactive IL-10, critical role of cytoplasmic tail in autocrine signaling. Proc. Natl. Acad. Sci. U.S.A. 96, 13938–13943 10.1073/pnas.96.24.13938 10570177 PMC24169

[B26] Correale J. Farez M. (2007). Asociation between parasite infection and immune responses in Multiple Sclerosis. Ann. Neurol. 61, 97–108 10.1002/ana.21067 17230481

[B27] Correale J. Farez M. (2009). Helminth antigens modulate immune responses in cells from Multiple Sclerosis patients through TLR2-dependent mechanisms. J. Immunol. 183, 5999–6012 10.4049/jimmunol.0900897 19812189

[B28] Correale J. Farez M. (2011). The impact of parasite infections on the course of multiple sclerosis. J. Neuroimmunol. 233, 6–11 10.1016/j.jneuroim.2011.01.002 21277637

[B29] Correale J. Farez M. Razzitte G. (2008). Helminth infections associated with Multiple Sclerosis induce regulatory B cells. Ann. Neurol. 64, 187–199 10.1002/ana.21438 18655096

[B30] Correale J. Fiol M. Gilmore W. (2006). The risk of relapses in multiple sclerosis during systemic infections. Neurology 67, 652–659 10.1212/01.wnl.0000233834.09743.3b 16870812

[B31] Correale J. Villa A. (2010). Role of CD8+ CD25+ Foxp3+ regulatory T cells in multiple sclerosis. Ann. Neurol. 67, 625–638 10.1002/ana.21944 20437560

[B32] Croxford J. L. Feldmann M. Chernajovsky Y. Baker D. (2001). Different therapeutic outcomes in experimental allergic encephalomyelitis dependent upon the mode of delivery of IL-10, a comparison of the effects of protein, adenoviral or retroviral IL-10 delivery into the Central Nervous System. J. Immunol. 166, 4124–4130 11238662 10.4049/jimmunol.166.6.4124

[B33] Danilowicz-Luebert E. O'Regan N. L. Stainfelder S. Hartmann S. (2011). Modulation of specific and allergic-related immune responses by helminthes. J. Biomed. Biotechnol. 2011, 821578 10.1155/2011/821578 22219659 PMC3248237

[B34] Donelly S. O'Neil S. M. Stack C. M. Robinson M. W. Turnbull L. Whitchurch C. Dalton J. P. (2010). Helminth cysteine proteases inhibit TRIF-dependent activation of macrophages via degradation of TLR3. J. Biol. Chem. 285, 3383–3392 10.1074/jbc.M109.060368 19923225 PMC2823461

[B35] Duddy M. Niino M. Adatia F. Hebert S. Freedman M. Atkins H. Kim H. J. Bar-Or A. (2007). Distinct effector cytokines profiles of memory and naïve human B cell subsets and implication in multiple sclerosis. J. Immunol. 178, 6092–6099 17475834 10.4049/jimmunol.178.10.6092

[B36] Elias D. Britton S. Aseffa A. Engers H. Akuffo H. (2008). Poor immunogenicity of BCG in helminth infected population is associated with increased *in vitro* TGF-beta production. Vaccine 26, 3897–3902 10.1016/j.vaccine.2008.04.083 18554755

[B37] Elliot D. E. Weinstock J. V. (2009). Helminth therapy: using worms to treat immune-mediated disease, in Pathogen-derived Immunomodualtory Molecules, ed Fallon P. G. (New York, NY; Landes Bioscience and Springer Science), 157–166

[B38] Ellmerich S. Mycko M. Takacs K. Waldner H. Wahid F. N. Boyton R. J. King R. H. Smith P. A. Amor S. Herlihy A. H. Hewitt R. E. Jutton M. Price D. A. Hafler D. A. Kuchroo V. K. Altmann D. M. (2005). High incidence of spontaneous disease in HLA-DR15 and TCR transgenic multiple sclerosis. J. Immunol. 174, 1938–1946 15699121 10.4049/jimmunol.174.4.1938

[B39] Everts B. Perona-Wright G. Smits H. H. Hokke C. H. van der Ham A. J. Fitzsimmons C. M. Doenhoff M. J. van der Bosch J. Mohrs K. Haas H. Mohrs M. Yazdanbakhsh M. Schramm G. (2009). Omega-1, a glycoprotein secreted by *Schistosoma mansoni* eggs drives Th2 responses. J. Exp. Med. 206, 1673–1680 10.1084/jem.20082460 19635864 PMC2722183

[B40] Everts B. Smits H. H. Hokke C. H. Yazdanbaksh M. (2010). Helminths and dendritic cells: sensing and regulating via pattern recognition receptors, Th2 and Treg responses. Eur. J. Immunol. 40, 1525–1537 10.1002/eji.200940109 20405478

[B41] Fillatreau S. Sweenie C. H. McGeachy M. J. Gary D. Anderton S. M. (2002). B cells regulate autoimmunity by provision of IL-10. Nat. Immunol. 3, 944–950 10.1038/ni833 12244307

[B42] Fleming J. O. Cook T. D. (2006). Multiple sclerosis and the hygiene hypothesis. Neurology 67, 2085–2086 10.1212/01.wnl.0000247663.40297.2d 17159130

[B43] Franchiotta D. Salvetti M. Lolli F. Serafini B. Aloisi F. (2008). B cells and multiple sclerosis. Lancet Neurol. 7, 852–858 10.1016/S1474-4422(08)70192-3 18703007

[B44] Frohman E. M. Filippi M. Stuve O. Waxman S. G. Corboy J. Phillips J. T. Lucchinetti C. Wilken J. Karandikar N. Hemmer N. Monson J. De Keyser J. Hartung H. Steinman L. Oksenberg J. Cree B. A. Hauser S. Racke M. K. (2005). Characterizing the mechanisms of progression in multiple sclerosis: evidence and new hypothesis for future directions. Arch. Neurol. 62, 1345–1356 10.1001/archneur.62.9.1345 16157741

[B45] Frohman E. M. Racke M. K. Raine C. S. (2006). Multiple sclerosis-the plaque and its pathogenesis. N. Engl. J. Med. 354, 942–955 10.1056/NEJMra052130 16510748

[B46] Furlan R. Cuomo C. Martino G. (2009). Animal models of multiple sclerosis. Methods Mol. Biol. 549, 157–173 10.1007/978-1-60327-931-4_11 19378202

[B47] Gay N. J. Gangloff M. (2007). Structure and function of Toll receptors and their ligands. Annu. Rev. Biochem. 76, 141–165 10.1146/annurev.biochem.76.060305.151318 17362201

[B48] Geijtenbeek T. B. van Vliet S. J. Koppel E. A. Sanchez-Hernandez M. Vandenbroucke-Grauls C. M. Appelmek B. van Kooyk Y. (2003). Mycobacteria target DC-SIGN to suppress dendritic cells function. J. Exp. Med. 197, 7–17 12515809 10.1084/jem.20021229 PMC2193797

[B49] Genain C. P. Canella B. Hauser S. Raine C. S. (1999), Identification of autoantiboides associated with myelin damage in multiple sclerosis. Nat. Med. 5, 170–175 10.1038/5532 9930864

[B50] Godfrey D. I. Kroneneberg M. (2004). Going both ways: immune regulation via CD1d-dependent NKT cells. J. Clin. Invest. 114, 1379–1388 10.1172/JCI23594 15545985 PMC525753

[B51] Gomez-Escobar N. Gregoory W. F. Maizels R. M. (2000). Identification of tgh-2 a filarial nematode homolog of *Caenorhabditis elegans* daf-7 and human transforming growth factor β, expressed in microfilarial and adult stages of *Brugia malayi*. Infect. Immun. 68, 6402–6410 10.1128/IAI.68.11.6402-6410.2000 11035752 PMC97726

[B52] Goodridge H. S. Marshall F. A. Else K. J. Houston K. M. Egan C. Al-Ryami L. Liew F. Y. Harnett W. Harnett M. M. (2005). Immunomodulation via novel use of TLR4 by the filiarial nematode phosphorylcholine-containing secreted product, ES-62. J. Immunol. 174, 284–293 15611251 10.4049/jimmunol.174.1.284

[B53] Goverman J. (2009). Autoimmune T cell responses in the central nervous system. Nat. Rev. Immunol. 9, 393–407 10.1038/nri2550 19444307 PMC2813731

[B54] Grainger J. R. Smith K. A. Hewitson J. P. McSorley H. J. Harcus Y. Filbey K. J. Finney C. A. Greenwood E. J. (2010). Helminth secretions induce *de novo* T cell Foxp3 expression and regulatory function through the TGF-β pathways. J. Exp. Med. 207, 2331–2341 10.1084/jem.20101074 20876311 PMC2964568

[B55] Greter M. Heppner F. L. Lemos M. P. Odermatt B. M. Goebels N. Laufer T. Noelle R. J. Becher B. (2005). Dendritic cells permit immune invasion of the CNS in an animal model of multiple sclerosis. Nat. Med. 11, 328–334 10.1038/nm1197 15735653

[B56] Klotz C. Ziegler T. Danilowicz-Luebert E. Hartmann S. (2011). Cystatins of parasitic organisms. Adv. Exp. Med. Biol. 712, 208–221 10.1007/978-1-4419-8414-2_13 21660667

[B58a] Hammad H. Charbonnier A. S. Duez C. Jacquet A. Stewart G. A. Tonnel A. B. Pastel J. (2001). Th2 polariztion by Der p1-pulsed monocyte derived dendritic cells is due to the allergic status of the donors. Blood 98, 1135–1141 11493462 10.1182/blood.v98.4.1135

[B57] Handel A. E. Lincoln M. R. Ramagopalan S. V. (2011). Of mice and men: experimental autoimmune encephalomyelitis and multiple sclerosis. Eur. J. Clin. Invest. 41, 1254–1258 10.1111/j.1365-2362.2011.02519.x 21418205

[B58] Harnett W. Harnett M. M. (2009). Immunomodulatory activity and therapeutic potential of the filarial nematode secreted product, ES-62, in Pathogen-derive Immunomodualtory Molecules, ed Fallon P. G. (New York, NY; Landes Bioscience and Springer Science), 88–92 10.1007/978-1-4419-1601-3_7 20054977

[B59] Hartmann S. Kyewski B. Sonnenburg B. Lucius R. (1997). A filarial cysteine proteinase inhibitor down-regulates T cell proliferation and enhances interleukin-10 production. Eur. J. Immunol. 27, 2253–2260 10.1002/eji.1830270920 9341767

[B60] Harrington L. E. Hatton R. D. Mangan P. R. Turner H. Murphy T. L. Murphy K. M. Weaver C. T. (2005). Interleukin 17-producing CD4+ effector T cells develop via a lineage distinct from T helper type 1 and 2 lineages. Nat. Immunol. 6, 1123–1132 10.1038/ni1254 16200070

[B61] Hartgers F. C. Obeng B. B. Voskamp A. Larbi I. A. Amoah A. S. Luty A. J. F. Boakye D. Yazdanbakhsh M. (2008). Enhanced Toll-like receptor responsiveness associated with mitogen-activated protein kinase activation in *Plasmodium falciparum*-infected children. Infect. Immun. 76, 5149–5157 10.1128/IAI.01579-07 18710867 PMC2573356

[B62] Holman D. W. Klein R. S. Ransohoff R. M. (2011). The blood-brain-barrier, chemokines and multiple sclerosis. Biochim. Biophys. Acta 182, 220–230 10.1016/j.bbadis.2010.07.019 20692338 PMC3005102

[B63] Imai S. Tezuka H. Fujita K. (2001). A factor of inducing IgE from filarial parasite prevents insulin-dependent diabetes mellitus in nonobese diabetic mice. Biochem. Biophys. Res. Commun. 286, 1051–1058 10.1006/bbrc.2001.5471 11527407

[B64] Jankovic D. Steinfelder S. Kullberg M. C. Sher A. (2006). Mechanisms underlying helminth-induced Th2 polarization: default, negative or positive pathways. Chem. Immunol. Allergy 90, 65–81 10.1159/000088881 16210903

[B65] Kamda J. D. Singer S. M. (2009). Phosphoinositide 3-kinase-dependent inhibition of dendritic cells interleukin-12 production by *Giardia lamblia*. Infect. Immun. 77, 685–693 10.1128/IAI.00718-08 19047410 PMC2632045

[B66] Kane C. M. Cervi L. Sun J. McKee A. S. Masek K. S. Shapiara S. Hunter C. A. Pearce E. J. (2004). Helminth antigens modulate TLR-initiated dendritic cell activation. J. Immunol. 173, 7454–7461 15585871 10.4049/jimmunol.173.12.7454

[B67] Kane C. M. Jung E. Pearce E. J. (2008). *Schistosoma mansoni* egg antigen-mediated modulation of Toll-like receptor (TLR)-induced activation occurs in dependently TLR2, TLTR4, and MyD88. Infect. Immun. 76, 5754–5759 10.1128/IAI.00497-08 18824534 PMC2583582

[B68] Kawai T. Akira S. (2005). Pathogen recognition with Toll-like receptors. Curr. Opin. Immunol. 17, 338–344 10.1016/j.coi.2005.02.007 15950447

[B69] Kennedy M. K. Torrance D. S. Picha K. S. Mohler K. M. (1992). Analysis of cytokine mRNA expression in the central nervous system of mice with experimental autoimmune encephalomyelitis reveals that IL-10 mRNA expression correlates with recovery. J. Immunol. 149, 2496–2505 1527389

[B70] Korn T. Betelli M. Oukka M. Kuchroo V. J. (2009). IL-17 and TH17 cells. Annu. Rev. Immunol. 27, 485–517 10.1146/annurev.immunol.021908.132710 19132915

[B71] Kuijk L. M. van Die I. (2010). Worms to rescue: can worm Glycans protect from autoimmune diseases? Life 62, 303–312 10.1002/iub.304 20101628

[B72] La Flamme A. C. Ruddenklau K. Bäckström B. T. (2003). Schistosomiasis decreases central nervous inflammation and alters the progression of experimental autoimmune encephalomyelitis. Infect. Immun. 71, 4996–5004 10.1128/IAI.71.9.4996-5004.2003 12933842 PMC187318

[B73] Lampropoulou V. Hoeghlig K. Roch T. Neves P. Calderón-Gomez C. Sweenie H. Hao Y. Freitas A. A. Steinhoff U. Anderton S. M. Fillatreau S. (2008). TLR-B activated B cells suppress T-cell mediated autoimmunity. J. Immunol. 180, 4763–4773 18354200 10.4049/jimmunol.180.7.4763

[B74a] Lassmann H. (2008). Mechanims of inflammation induced tissue injury in multiple sclerosis. J. Neurol. Sci. 274, 45–47 10.1016/j.jns.2008.04.003 18495163

[B74] Lassmann H. Bruck W. Lucchinetti C. (2001). Heterogeneity of multiple sclerosis pathogenesis: implications for diagnosis and therapy. Trends Mol. Med. 7, 115–121 10.1016/S1471-4914(00)01909-2 11286782

[B75] Layland L. E. Rad R. Wagner H. da Costa C. U. (2007). Immunopathology of schistosomiasis is controlled by antigen-specific regulatory T cells primed in the presence of TLR2. Eur. J. Immunol. 37, 2174–2184 10.1002/eji.200737063 17621370

[B76] Lorenz E. Mira J. P. Cornish K. L. Arbour N. C. Schwartz D. A. (2000). A novel polymorphism in the Toll-like receptor 2 gene and its potential association with staphylococcal infection. Infect. Immun. 68, 6398–6401 10.1128/IAI.68.11.6398-6401.2000 11035751 PMC97725

[B77] Lotz M. Gütle D. Walther S. Menard S. Bogdan C. Hornef M. W. (2006). Postnatal acquisition of endotoxin tolerance in intestinal epithelial cells. J. Exp. Med. 203, 973–984 10.1084/jem.20050625 16606665 PMC2118301

[B78] Magalhaes K. G. Almeida P. E. Atella G. C. Maya-Monteiro C. M. Castro-Faria-Neto H. C. Pelajo-Machado M. Lenzi H. L. Bozza M. T. Bozza P. T. (2010). Schistosomal-derived lysophosphatidylcholine are involved in eosinophil activation and recruitment through Toll-like receptor-2-dpendent mechanisms. J. Infect. Dis. 202, 1369–1379 10.1086/656477 20863227

[B79] Magliozzi R. Howell O. Vora A. Serafini B. Nicholas R. Puopolo M. Reynolds R. Aloisi F. (2007). Meningeal-B cell follicles in secondary progressive multiple sclerosis associated with early onset of disease and severe cortical pathology. Brain 130, 1089–1104 10.1093/brain/awm038 17438020

[B80] Mahad D. J. Ziabreva I. Campbell G. Lax N. White K. Hanson P. S. Lassmann H. Turnbull D. M. (2009). Mitochondrial changes within axons in multiple sclerosis. Brain 132, 1161–1174 10.1093/brain/awp046 19293237 PMC3605917

[B81] Manoury B. Gregory W. F. Maizels R. M. Watts C. (2001). Bm-CPI-2, a cystatin homolog secreted by the filarial parasite *Brugia malayi*, inhibits class II MC-restricted antigen processing. Curr. Biol. 11, 447–451 10.1016/S0960-9822(01)00118-X 11301256

[B82] Marrie R. A. (2004). Environmental risk factors in multiple sclerosis. Lancet Neurol. 3, 709–718 10.1016/S1474-4422(04)00933-0 15556803

[B83] Marta M. Andersson A. Isaksson M. Kampe O. Lobell A. (2008). Unexpected regulatory roles of TLR4 and TLR9 in experimental autoimmune encephalomyelitis. Eur. J. Immunol. 38, 565–575 10.1002/eji.200737187 18203139

[B84] Marta M. Meier U. C. Lobell A. (2009). Regulation of autoimmune encephalomyelitis by toll-like receptors. Autoimmunity Rev. 8, 506–509 10.1016/j.autrev.2009.01.006 19211042

[B85] Marthur R. K. Awasthi A. Wadhone P. Ramanamurthy B. Saha B. (2004). Reciprocal CD40 signals through p38MAPK and ERK-1/2 induce counteracting immune responses. Nat. Med. 10, 540–544 10.1038/nm1045 15107845

[B86] Mastisz C. E. McDougall J. J. Sharkey K. A. McKay D. M. (2011). Helminth parasites and the modulation of joint inflammation. J. Paras. Res. 2011, 942616 10.1155/2011/942616 21584243 PMC3092582

[B87] McFarland H. F. Martin R. (2007). Multiple Sclerosis: a complicated picture of autoimmunity. Nat. Immunol. 8, 913–919 10.1038/ni1507 17712344

[B88] Medana I. Martinic M. A. Wekerle H. Neumann H. (2001). Transection of major histocompatibility complex class I-induced neuritis by cytototxic T lymphocytes. Am. J. Pathol. 159, 809–815 10.1016/S0002-9440(10)61755-5 11549572 PMC1850471

[B89] Meyer-Wentrup F. Cambi A. Joosten B. Looman M. W. de Vries I. Figdor C. G. Adema G. J. (2009). DCIR is endocytosed into human dendritic cells and inhibits TLR-8 mediated cytokine production. J. Leukoc. Biol. 85, 518–525 10.1189/jlb.0608352 19028959

[B90] Mix E. Meyer-Reienecker H. Hartung H. P. Zettl U. K. (2010). Animal models for multiple sclerosis – potential and limitations. Progr. Neurobiol. 92, 386–404 10.1016/j.pneurobio.2010.06.005 20558237 PMC7117060

[B91] Monney L. Sabatos C. A. Gaglia J. L. Ryu A. Waldner H. Chernova T. Manning S. Greenfiled E. A. Coyle A. J. Sobel R. A. Freeman G. J. Kuchroo V. J. (2002). Th1-specific cell surface protein Tim-3 regulates macrophage activation and severity of an autoimmune disease. Nature 415, 536–541 10.1038/415536a 11823861

[B92] Neumann H. Medana I. M. Bauer J. Lassmann H. (2002). Cytotoxic T lumphocytes in autoimmune and degenerative CNS diseases. Trends Neurosci. 25, 313–319 10.1016/S0166-2236(02)02154-9 12086750

[B93] O'Garra A. Barrat F. J. Castro A. G. Vicari A. Hawryloowicz C. (2008). Strategies for use of IL-10 or its antagonists in human disease. Ann. Rev. Immunol. 223, 114–131 10.1111/j.1600-065X.2008.00635.x 18613832

[B94] Oksenberg J. R. Baranzini S. E. (2010). Multiple sclerosis genetics-is the glass half full, or half empty? Nat. Rev. Neurol. 6, 429–437 10.1038/nrneurol.2010.91 20625377

[B95] O'Neill L. A. Bowie A. G. (2007). The family of five: TIR-domain-containing adaptors in Toll-like receptor signaling. Nat. Rev. Immunol. 7, 353–364 10.1038/nri2079 17457343

[B96] O'Neill L. A. J. (2008). Primer: Toll-like receptor signaling pathways what do rheumatologist need to know? Nat. Clin. Pract. Rheum. 4, 319–327 10.1038/ncprheum0802 18446139

[B97] Oshiro T. M. Macedo M. S. Macedo-Soares M. F. (2005). Anti-inflammatory activity of PAS-1 a protein component of *Ascaris suum*. Inflamm. Res. 54, 17–21 10.1007/s00011-004-1316-7 15723200

[B98] Piccio L. Rossi B. Scarpini E. Laudanna C. Giagulli C. Issekutz A. C. Vestweber D. Butcher E. C. Constantin G. (2002). Molecular mechanisms involved in lymphocyte recruitment in inflamed brain microvessels: critical roles for P-selectin glycoprotein ligand-1 and heterotrimeric G(i)-linked receptors. J. Immunol. 168, 1940–1949 11823530 10.4049/jimmunol.168.4.1940

[B99] Pöllinger B. Krishnamoorthy G. Berer K. Lassmann H. Bösl M. R. Dunn R. Domingues H. S. Holz A. Kurschus F. C. Wekerle H. (2009). Spontaneous relapsing-remitting EAE in the SJL/J mouse:MOG-reactive transgenic T cells recruited endogenous MOG-specific B cells. J. Exp. Med. 206, 1303–1316 10.1084/jem.20090299 19487416 PMC2715069

[B100] Prinz M. Garbe F. Schmidt H. Mildner A. Gutcher I. Wolter K. Piesche M. Schroers R. Weiss E. (2006). Innate immunity mediated by TLR9 modulates pathogenicity in an animal model of multiple sclerosis. J. Clin. Invest. 116, 456–464 10.1172/JCI26078 16440059 PMC1350999

[B101] Racke M. K. Drew P. D. (2009). Toll-like receptors in multiple sclerosis. Curr. Top. Microbiol. Immunol. 336, 155–168 10.1007/978-3-642-00549-7_9 19688333 PMC3064511

[B102] Reyes J. L. Espinoza-Jinménez A. F. Gonzalez M. I. Verdin L. Terrazas L. I. (2011). Taenia crassiceps infection aborgates experimental autoimmune encephalomyelitis. Cell. Immunol. 267, 77–87 10.1016/j.cellimm.2010.11.006 21185554

[B103] Rincon M. Enslen H. Raingeaud J. Recht M. Zapton T. Su S. M. Penix L. A. Davis R. J. Flavell R. A. (1998). Interferon-γ expression by Th1 effector T cells mediated by p38MAP kinase signaling pathway. EMBO J. 17, 2817–2829 10.1093/emboj/17.10.2817 9582275 PMC1170622

[B104] Rott O. Fleischer B. Cash E. (1994). Interleukin-10 prevents experimental allergic encephalomyelitis in rats. Eur. J. Immunol. 24, 1434–1440 10.1002/eji.1830240629 7515815

[B105] Serafini B. Rosicarelli B. Magliozzi R. Stigliano E. Capello E. Mancardi G. L. Aloisi F. (2006). Dendritic cells in multiple sclerosis lesions: maturation stage, myelin uptake, and interaction with proliferating T cells. J. Neuropathol. Exp. Neurol. 65, 124–141 10.1097/01.jnen.0000199572.96472.1c 16462204

[B106] Sewell D. Qing Z. Reinke E. Elliot D. Weinstock J. Sandor M. Fabry Z. (2003). Immunomodualtion of experimental autoimmune encephalomyelitis by helminth ova immunization. Int. Immunol. 15, 59–69 10.1093/intimm/dxg012 12502726

[B107] Smith K. J. Lassmann H. (2002). The role of nitric oxide in multiple sclerosis. Lancet Neurol. 1, 232–241 12849456 10.1016/s1474-4422(02)00102-3

[B108] Steinman L. (1999). Assesment of animal models for MS and demyelinating disease in the design of rational therapy. Neuron 24, 511–514 10.1016/S0896-6273(00)81107-1 10595504

[B109] Strommes I. M. Cerretti L. M. Liggit D. Harris R. A. Goverman J. M. (2008). Differential regulation of central nervous system autoimmunity by T(H)1 and T(H)17 cells. Nat. Med. 14, 337–342 10.1038/nm1715 18278054 PMC2813727

[B110] van der Kleij D. Latz E. Brouwers J. F. H. M. Kruize Y. C. M. Schmitz M. Kurt-Jones E. A. Espevik T. de Jong E. C. Kapsenberg M. L. Golenbock D. T. Tielens A. G. Yazdanbakhsh M. (2002). A novel host-parasite lipid cross-talk. J. Biol. Chem. 277, 48122–48129 10.1074/jbc.M206941200 12359728

[B111] van Riet E. Retra K. Adegnika A. A. Vieira R. Tielens A. G. Lell B. Issifou S. Hartgers F. C. Rimmelzwaan G. F. Kremsner P. G. Yazdanbakhsh M. (2007). Cellular and humoral responses to influenza in Gabonese children living in rural and semi-urban areas. J. Infect. Dis. 196, 1671–1678 10.1086/522010 18008252

[B112] van Riet E. Retra K. Adegnika A. A. Jol-van der Zijde C. M. Uh H. W. Lell B. Issifou S. Kremsner P. G. Yazdanbakhsh M. van Tol M. J. Hartgers F. C. (2008). Cellular and humoral responses to tetanus vaccination in Gabonese children. Vaccine 26, 3690–3695 10.1016/j.vaccine.2008.04.067 18539369

[B113] Velupillai P. Secor W. E. Horauf Harm A. M. (1997). B-1 cell (CD5+B220+) outgrowth in murine schistosomiasis is genetically restricted and is largely due to activation by polylactosamine sugars. J. Immunol. 158, 338–344 8977208

[B114] Viglietta V. Baecher-Allan C. Weiner H. L. Hafler D. A. (2004). Loss of functional suppression by CD4+CD25+ regulatory T cells in patients with multiple sclerosis. J. Exp. Med. 199, 971–979 10.1084/jem.20031579 15067033 PMC2211881

[B115] Walsh K. P. Brady M. T. Finlay C. M. Boon L. Mills K. H. G. (2009). Infection with helminth parasite attenuates autoimmunity through TGF-β-mediated suppression of Th17 and Th1 responses. J. Immunol. 183, 1577–1586 10.4049/jimmunol.0803803 19587018

[B116] Watters T. M. Kenny E. F. O'Neil L. A. (2007). Structure, function and regulation of the Toll/IL-1 receptor adaptor proteins. Immunol. Cell Biol. 85, 411–419 10.1038/sj.icb.7100095 17667936

[B117] Weiner H. L. (2004). Multiple Sclerosis is an inflammatory T-cell mediated autoimmune disease. Arch. Neurol. 61, 1613–1615 10.1001/archneur.61.10.1613 15477521

[B118] Weis W. I. Taylor M. E. Drickamer K. (1998). The C-type lectin superfamily in the immune system. Immunol. Rev. 163, 19–34 9700499 10.1111/j.1600-065x.1998.tb01185.x

[B119] Werner P. Pitt D. Raine C. S. (2001). Multiple sclerosis: an altered glutamate homeostasis in lesions correlates with oligodendrocyte and axonal damage. Ann. Neurol. 50, 169–180 11506399 10.1002/ana.1077

[B120] West A. P. Koblansky A. A. Gosh S. (2006). Recognition and signaling by toll-like receptors. Ann. Rev. Cell Dev. Biol. 22, 409–437 10.1146/annurev.cellbio.21.122303.115827 16822173

[B121] Yednock T. A. Cannon C. Fritz L. C. Sanchez-Madrid F. Steinman L. Karin N. (1992). Prevention of experimental autoimmune encephalomyelitis by antibodies against alpha 4 beta 1 integrin. Nature 356, 63–66 10.1038/356063a0 1538783

[B122] Yu L. Wang L. Chen S. (2010). Endogenous Toll-like receptor ligands and their biological significance. J. Cell Mol. Med. 14, 2592–2603 10.1111/j.1582-4934.2010.01127.x 20629986 PMC4373479

